# Supplementary material for: A conserved grain-associated immunosuppressive niche in Sudanese patients with mycetoma
Source: PLoS Negl Trop Dis. 2026 Aug 17;20(8):e0014230. doi: 10.1371/journal.pntd.0014230 (PMC13497247; doi:10.1371/journal.pntd.0014230)
Supplement: Supplementary file 1 [file pntd.0014230.s004.pdf]

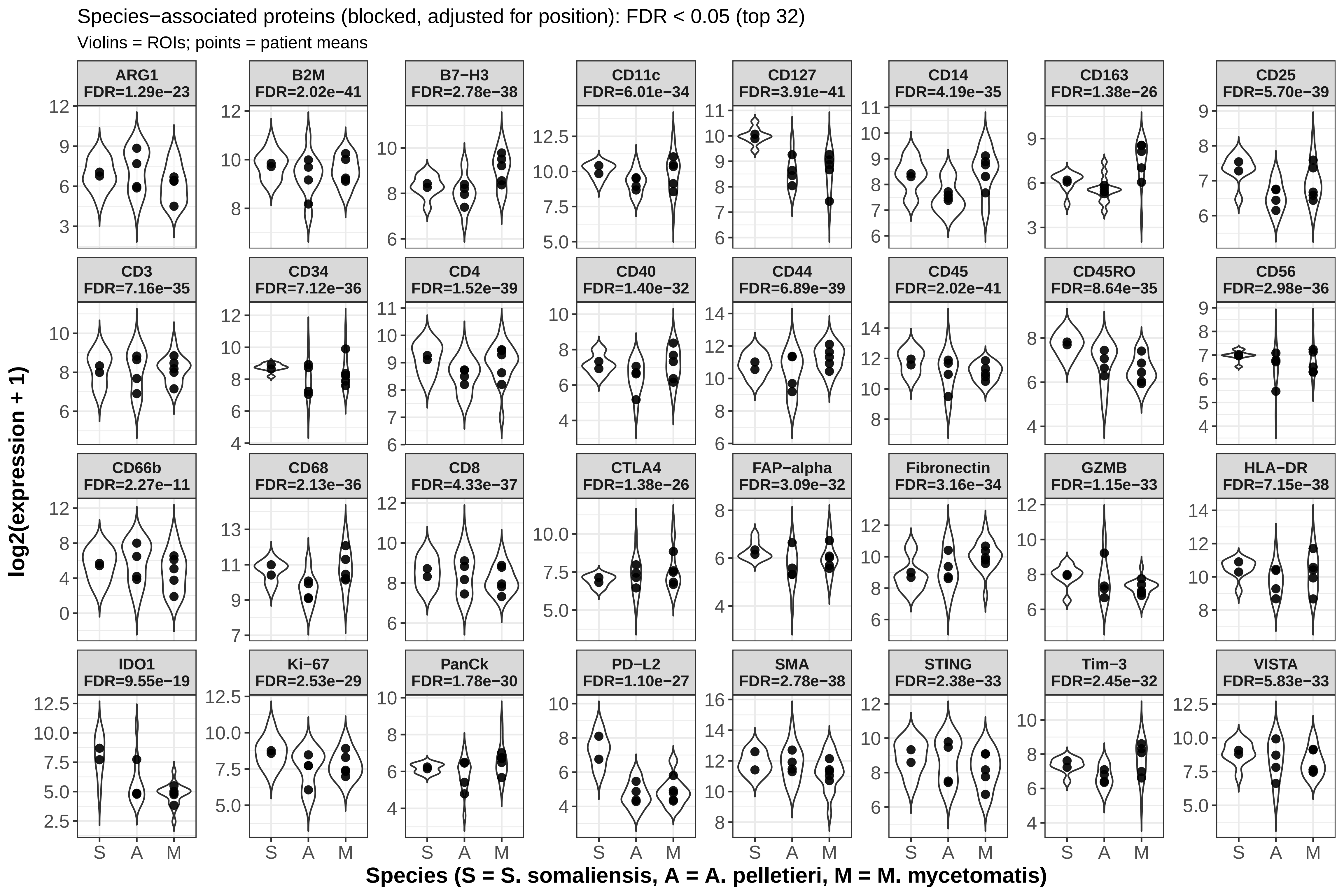

Species−associated proteins (blocked, adjusted for position): FDR < 0.05 (top 32)
Violins = ROIs; points = patient means
ARG1 FDR=1.29e−23
B2M FDR=2.02e−41
B7−H3 FDR=2.78e−38
CD11c FDR=6.01e−34
CD127 FDR=3.91e−41
CD14 FDR=4.19e−35
CD163 FDR=1.38e−26
CD25 FDR=5.70e−39
CD3 FDR=7.16e−35
CD34 FDR=7.12e−36
CD4 FDR=1.52e−39
CD40 FDR=1.40e−32
CD44 FDR=6.89e−39
CD45 FDR=2.02e−41
CD45RO FDR=8.64e−35
CD56 FDR=2.98e−36
CD66b FDR=2.27e−11
CD68 FDR=2.13e−36
CD8 FDR=4.33e−37
CTLA4 FDR=1.38e−26
FAP−alpha FDR=3.09e−32
Fibronectin FDR=3.16e−34
GZMB FDR=1.15e−33
HLA−DR FDR=7.15e−38
IDO1 FDR=9.55e−19
Ki−67 FDR=2.53e−29
PanCk FDR=1.78e−30
PD−L2 FDR=1.10e−27
SMA FDR=2.78e−38
STING FDR=2.38e−33
Tim−3 FDR=2.45e−32
VISTA FDR=5.83e−33
log2(expression + 1)
S A M
Species (S = S. somaliensis, A = A. pelletieri, M = M. mycetomatis)
