# Supplementary material for: A conserved grain-associated immunosuppressive niche in Sudanese patients with mycetoma
Source: PLoS Negl Trop Dis. 2026 Aug 17;20(8):e0014230. doi: 10.1371/journal.pntd.0014230 (PMC13497247; doi:10.1371/journal.pntd.0014230)
Supplement: Supplementary file 2 [file pntd.0014230.s002.docx]

**Figure S1. Representative patient histology (H&E).**

For patient details, see Table S1. Scale bar: 200μm
