# Supplementary material for: A conserved grain-associated immunosuppressive niche in Sudanese patients with mycetoma
Source: PLoS Negl Trop Dis. 2026 Aug 17;20(8):e0014230. doi: 10.1371/journal.pntd.0014230 (PMC13497247; doi:10.1371/journal.pntd.0014230)
Supplement: Supplementary file 4 [file pntd.0014230.s005.docx]

C

A

B

**Figure S4. Correlations plots for Far ROIs**

**A.** Heat map of protein correlation across all Far ROIs using data set enriched in near ROIs. **B, C**. Correlations between CD66b and ARG1 (B) and VISTA (C) in far ROIs.
