# Supplementary material for: A conserved grain-associated immunosuppressive niche in Sudanese patients with mycetoma
Source: PLoS Negl Trop Dis. 2026 Aug 17;20(8):e0014230. doi: 10.1371/journal.pntd.0014230 (PMC13497247; doi:10.1371/journal.pntd.0014230)
Supplement: Supplementary file 5 [file pntd.0014230.s006.docx]

*

**Figure S5. IHC staining for CD15 and SIGLEC-8**

Section from patient P27 showing rare SIGLEC-8^+^ cells (yellow) beyond the peri-grain accumulation of CD15^+^ myeloid cells (red), with DAPI counterstain (white). Weak autofluorescence is also observed in the grain (*). Scale bar 100um for main figure and 10um for insert (shown left). Absence of CD15 on SIGLEC-8^+^ cells is suggestive of tissue eosinophils.
