# Supplementary material for: A conserved grain-associated immunosuppressive niche in Sudanese patients with mycetoma
Source: PLoS Negl Trop Dis. 2026 Aug 17;20(8):e0014230. doi: 10.1371/journal.pntd.0014230 (PMC13497247; doi:10.1371/journal.pntd.0014230)
Supplement: Supplementary file 6 [file pntd.0014230.s008.docx]

**Reviewer #1**

*Please avoid including the results or conclusions in the introduction section.
Please clearly state the objectives on the last paragraph of the introduction*.

We have amended the last paragraph of the Introduction as requested (**lines 112-114**).

*In the methods include country and city of the devices, software, and stains employed.*

We thank the reviewer for noting these omissions which have now been corrected (**Methods**)

*The design is appropriate and the population clearly stated. The sample size is small but that would be expected for such a rare disease. Ethical standards were followed. The statistical analysis is complex but adequate. The analysis matches the methods section and are clearly presented. The figures are of great quality.*

We thank the reviewer for these kind comments regarding the quality of the analysis and presentation.

*Please include in this section what VISTA means instead of in the discussion section.*

We have now included the full name for VISTA at first use (**line 178**).

*Please include if any of the patients included had comorbidities (HIV, malnutrition, diabetes, etc.).*

None of the patients was HIV positive, diabetic or malnourished. This information has been added to the text (**line 138-139**).

*Please replace “chemotherapeutic agents” with antibiotics in line 314.*

Corrected as requested (**line 324**).

*Please replace “benign” with a more appropriate term.*

We have replaced the sentence containing “benign” with something more appropriate (**line 324-325**)

**Reviewer #2**

*The study objectives are generally clear, aiming to characterize the spatial immune microenvironment in mycetoma; however, a clearly defined, testable primary hypothesis is lacking, and the work is better framed as exploratory.*

We agree and had noted that the study and analyses were exploratory in the Introduction and on five other occasions in the Abstract, Results and Discussion. We have now also added the word “exploratory” to the final summary paragraph of the paper to further emphasize this point (**line 398**).

*The study design is appropriate for spatial proteomic analysis, but the inclusion of only 11 out of 28 patients based on grain visibility and the use of manually selected ROIs introduce potential selection bias that should be more explicitly addressed.*

We have now noted this important point in the limitations section of the Discussion (**lines 388-390**)

*Ethical and regulatory requirements appear to be adequately met, with appropriate approvals and informed consent obtained. The analytical approach is appropriate and robust for the study design; however, the exploratory nature and limited sample size should be more explicitly acknowledged when interpreting the findings.*

We thank the reviewer for these kind comments on the analytical approach. As noted above, we have included the word exploratory on multiple occasions throughout the manuscript, including in the limitations paragraph (line 387).

*The conclusions are generally supported by the data, particularly the identification of a peri-grain immune microenvironment enriched in CD66b⁺ARG1⁺VISTA⁺ cells. The limitations are acknowledged but could be more explicitly discussed, especially regarding sample size and potential bias.*

We have addressed these comments above.

**Reviewer #3**:

*As a neglected tropical disease, effective treatment of mycetoma requires a better understanding regarding the pathophysiology and virulence factors driving this disease. Therefore, the concept behind this manuscript and employing multiplex microscopy as well as spatial proteome analysis could address an interesting question on 1) the architecture and positioning of immune cells near the granuloma structure and 2) effector proteins shaping the immune cell response. Despite the importance and novelty, a few considerations and some experimental set-ups are required to tackle the speculations.*

We thank the reviewer for appreciating the value of the study and making the suggestions below.

*1. CD66b is a degranulation marker expressed by both neutrophils and eosinophils. Since eosinophils are long known to be involved and drive anti-inflammatory responses, it would be prudent that authors differentiate the two using microscopy. If neutrophils, therefore the near ROI should be CD15+/CD16+/CD14- and if eosinophils sigelc-8, CCR3, and IL-5Rα should be positive.*

We thank the reviewer for this comment. Examination of H&E sections indicates that eosinophils are scarce in these biopsies and not associated with grains. We have also confirmed this observation by IHC staining for CD15 in combination with Siglec-8. These data are included as additional text and supplementary data (**lines 284-290** and **348-351**, **new** **Fig. S5**) and show that the cells enriched proximal to grains are CD15^+^ and Siglec-8^-^. In contrast Siglec-8^+^ eosinophils were rare, expressed little or no CD15 (in keeping with tissue residency) and located outside the peri-grain niche.

*2. Authors are required to provide further proof that indeed the environment is immunosuppressive given that the major outcome of this manuscript, which is indeed interesting, is that the near ROI is an immunosuppressive niche. Therefore, I would suggest determining the expression of IL4, IL13, and IL14 either using ELISA or using microscopy. The latter might be more interesting since it could show if those cytokines are colocalized with CD66b.*

It is challenging (in any field) to generate proof that a specific spatial niche is immunosuppressive based solely on FFPE sections. For these patients, neither blood nor further tissue is available. We have therefore based our conclusions purely on phenotypic marker expression and the wealth of published data that associates these markers with an immunosuppressive microenvironment. Whilst ARG1 is IL-4 inducible and VISTA is often upregulated in an M2-like environment, these are upstream events that drive ARG1 and VISTA expression and are not directly linked to the immunosuppression of T cell function mediated by ARG1 and VISTA. Hence, staining for these cytokines would not address the question raised by the reviewer. A more in-depth exploration of the expression of T cell activation states would be required and we believe this is beyond the scope of this exploratory study.

*3. Although it’s possible to infer that this immunosuppressive response is driven by the pathogen, authors should note that 1) the involvement of mycetoma in driving this response has yet to be fully confirmed and validated, and 2) this might be an appropriate immune response to the tissue destruction created by the grain structure and therefore maintaining homeostasis and healing. The latter is also possible since based on Figure 2, the expression of fibronectin and SMA are significantly lower in near compared to far ROI. Therefore, immunosuppressive immune response could be mounted to restore the shortage and restore homeostasis. But it also may imply that the proper immune cells restoring the homeostasis is not sufficient near ROI, which could question the very nature of the finding. That is why also inclusion of the alternatively activated cytokines- IL4/IL13/IL14- is important in this context.*

The reviewer highlights some important points. It is correct that our analysis does not address whether mycetoma organisms drive the response observed, and we made no assumptions with regard to this issue in the manuscript. For the sake of clarity, we have included an additional sentence in the Discussion on this issue (**lines 367-368**). We have also added a brief comment to the discussion to state that the presence of an immunosuppressive niche may be favourable in terms of limiting host pathology (**lines 383-385**). Whilst the reviewer speculates on the importance of an anti-inflammatory cytokine milieu (as also seen in the solid tumour microenvironment), cytokines are generally not included in spatial proteomics panels (due to their poor signal and tendency for diffusion). CD163 expression which often reflects an M2-like microenvironment was not enriched near grains, suggesting that cytokine expression may not be spatially regulated with respect to proximity to grains, but this would require further analysis, including through spatial transcriptomics. Many aspects of immunosuppression mediated by ARG1 and VISTA also operate in ways that do not directly involves anti-inflammatory cytokines.

*4. Why authors did not include inflammatory proteins, including IL6, IL1β, IFNγ, and TNFα?*

As the study was exploratory in nature, we used an immune-oncology protein panel to interrogate tissues. This, for the reasons given above, did not include cytokine specific antibodies. Based on the data derived from the proteomic analysis we focused attention on the cell types / phenotypes that showed skewed spatial distribution for subsequent validation by IHC. Whilst the reviewer lists a panel of inflammatory cytokines, many others could equally well be studied. As with anti-inflammatory cytokines, we suggest that analysis of cytokine expression might be better suited to a follow-on transcriptomic analysis and is, therefore, beyond the scope of this study.

*5. How the anti-inflammatory responses of actinomycetoma are reconciled with previous data suggesting that this complication is inflammatory as started by authors in the beginning of discussion?*

We cannot answer this question directly, but it is likely that spatial context is important. Our data are not at odds with a model whereby the overall environment is inflammatory but where specialised anti-inflammatory environments co-exist in specific spatial niches. Indeed, this is commonplace in complex diseases states ranging from cancer to autoimmune diseases and is perhaps one of the main advances made from the application of spatially resolved ‘omics technologies.

*Minor points
1. It would be prudent to provide microscopic images of all patients in supplementary files.*

Representative H&E images for each patient were included as Fig. S1.

*2. In the beginning of discussion section, since the data is premature, it is not recommended to mention that this CD66b hallmark of mycetoma is pathogenic, unless well-designed immunological studies are conducted to unravel this notion. The same is true for lines 364-366.*

We agree and have removed the word “pathogenic” from the text.
