# Supplementary material for: A conserved grain-associated immunosuppressive niche in Sudanese patients with mycetoma
Source: PLoS Negl Trop Dis. 2026 Aug 17;20(8):e0014230. doi: 10.1371/journal.pntd.0014230 (PMC13497247; doi:10.1371/journal.pntd.0014230)
Supplement: Peer review history

# Peer review: Decision Letter 0

**DOI:** 10.1371/journal.pntd.0014230.r001

**Authors:** Joseph Vinetz

30 Apr 2026

-->PNTD-D-26-00652

A conserved grain-associated immunosuppressive niche in Sudanese patients with mycetoma.

PLOS Neglected Tropical Diseases

Dear Dr. Kaye,

Thank you for submitting your manuscript to PLOS Neglected Tropical Diseases. After careful consideration, we feel that it has merit but does not fully meet PLOS Neglected Tropical Diseases's publication criteria as it currently stands. Therefore, we invite you to submit a revised version of the manuscript that addresses the points raised during the review process.

Please submit your revised manuscript within by Jun 29 2026 11:59PM. If you will need more time than this to complete your revisions, please reply to this message or contact the journal office at plosntds@plos.org. When you're ready to submit your revision, log on to https://www.editorialmanager.com/pntd/ and select the 'Submissions Needing Revision' folder to locate your manuscript file.

Please include the following items when submitting your revised manuscript:

* A letter that responds to each point raised by the editor and reviewer(s). You should upload this letter as a separate file labeled 'Response to Reviewers'. This file does not need to include responses to any formatting updates and technical items listed in the 'Journal Requirements' section below.

* A marked-up copy of your manuscript that highlights changes made to the original version. You should upload this as a separate file labeled 'Revised Manuscript with Track Changes'.

* An unmarked version of your revised paper without tracked changes. You should upload this as a separate file labeled 'Manuscript'.

If you would like to make changes to your financial disclosure, competing interests statement, or data availability statement, please make these updates within the submission form at the time of resubmission. Guidelines for resubmitting your figure files are available below the reviewer comments at the end of this letter.

We look forward to receiving your revised manuscript.

Kind regards,

Joseph M. Vinetz

Section Editor

PLOS Neglected Tropical Diseases

Joseph Vinetz

Section Editor

PLOS Neglected Tropical Diseases

Shaden Kamhawi

co-Editor-in-Chief

PLOS Neglected Tropical Diseases

orcid.org/0000-0003-4304-636XX

Paul Brindley

co-Editor-in-Chief

PLOS Neglected Tropical Diseases

orcid.org/0000-0003-1765-0002

**Journal Requirements:**

If the reviewer comments include a recommendation to cite specific previously published works, please review and evaluate these publications to determine whether they are relevant and should be cited. There is no requirement to cite these works unless the editor has indicated otherwise.

1) Please ensure that the CRediT author contributions listed for every co-author are completed accurately and in full.

At this stage, the following Authors/Authors require contributions: Mohamed Osman, Helen Ashwin, Grant Calder, Peter O'Toole, Sahar M. Bakhiet, Ahmed M. Musa, Paul M. Kaye, and Ahmed Hassan Fahal. Please ensure that the full contributions of each author are acknowledged in the "Add/Edit/Remove Authors" section of our submission form.

The list of CRediT author contributions may be found here: https://journals.plos.org/plosntds/s/authorship#loc-author-contributions

2) We do not publish any copyright or trademark symbols that usually accompany proprietary names, eg ©,  ®, or TM  (e.g. next to drug or reagent names). Therefore please remove all instances of trademark/copyright symbols throughout the text, including:

- ® on page: 8

- TM on page: 6.

3) Your manuscript's sections are not in the correct order.  Please amend to the following order: Abstract, Introduction, Results, Discussion, and Methods

4) Please upload all main figures as separate Figure files in .tif or .eps format. For more information about how to convert and format your figure files please see our guidelines:

https://journals.plos.org/plosntds/s/figures

5) Please amend your detailed Financial Disclosure statement. This is published with the article. It must therefore be completed in full sentences and contain the exact wording you wish to be published.

1) State the initials, alongside each funding source, of each author to receive each grant. For example: "This work was supported by the National Institutes of Health (####### to AM; ###### to CJ) and the National Science Foundation (###### to AM)."

2) State what role the funders took in the study. If the funders had no role in your study, please state: "The funders had no role in study design, data collection and analysis, decision to publish, or preparation of the manuscript."

3) If any authors received a salary from any of your funders, please state which authors and which funders..

If you did not receive any funding for this study, please simply state: u201cThe authors received no specific funding for this work.u201d

6) Some material included in your submission may be copyrighted. According to PLOS's copyright policy, authors who use figures or other material (e.g., graphics, clipart, maps) from another author or copyright holder must demonstrate or obtain permission to publish this material under the Creative Commons Attribution 4.0 International (CC BY 4.0) License used by PLOS journals. Please closely review the details of PLOS's copyright requirements here: PLOS Licenses and Copyright. If you need to request permissions from a copyright holder, you may use the PLOS Content Copyright Permission Form Please respond directly to this email and provide any known details concerning your material's license terms and permissions required for reuse, even if you have not yet obtained copyright permissions or are unsure of your material's copyright compatibility. Once you have responded and addressed all other outstanding technical requirements, you may resubmit your manuscript within Editorial Manager. Potential Copyright Issues:

i) We note that 1A is created through BioRender. Please confirm that you hold a Premium account and provide a pdf copy of the CC BY 4.0 Licence as provided by BioRender. For instructions on how to generate a CC BY 4.0 license for your figure, please see the guidelines here: https://help.biorender.com/hc/en-gb/articles/21282341238045-Publishing-in-open-access-resources

Please ensure the figure legend contains appropriate attribution text & a link to biorender.com

If you are using the free assets from BioRender, we are unable to publish these images as they are licenced under a stricter licence than CC BY 4.0. In this case we ask you to remove the BioRender images and replace them with open source alternatives.

See these open source resources you may use to replace images / clip-art:

- https://bioart.niaid.nih.gov/

- https://bioicons.com/

- https://healthicons.org/

- https://scidraw.io/

- https://reactome.org/icon-lib

- https://www.phylopic.org/images

- https://journals.plos.org/plosbiology/article?id=10.1371/journal.pbio.3002395

**Reviewers' Comments:**

Reviewer's Responses to Questions

**Key Review Criteria Required for Acceptance?**

As you describe the new analyses required for acceptance, please consider the following:

**Methods**

-Are the objectives of the study clearly articulated with a clear testable hypothesis stated?

-Is the study design appropriate to address the stated objectives?

-Is the population clearly described and appropriate for the hypothesis being tested?

-Is the sample size sufficient to ensure adequate power to address the hypothesis being tested?

-Were correct statistical analysis used to support conclusions?

-Are there concerns about ethical or regulatory requirements being met?

Reviewer #1: Please avoid including the results or conclusions in the introduction section.

Please clearly state the objectives on the last paragraph of the introduction.

In the methods include country and city of the devices, software, and stains employed.

The design is appropriate and the population clearly stated. The sample size is small but that would be expected for such a rare disease. Ethical standards were followed. The statistical analysis is complex but adequate.

Reviewer #2: The study objectives are generally clear, aiming to characterize the spatial immune microenvironment in mycetoma; however, a clearly defined, testable primary hypothesis is lacking, and the work is better framed as exploratory. The study design is appropriate for spatial proteomic analysis, but the inclusion of only 11 out of 28 patients based on grain visibility and the use of manually selected ROIs introduce potential selection bias that should be more explicitly addressed. Ethical and regulatory requirements appear to be adequately met, with appropriate approvals and informed consent obtained.

Reviewer #3: Yes. See detailed comments.

**********

**Results**

-Does the analysis presented match the analysis plan?

-Are the results clearly and completely presented?

-Are the figures (Tables, Images) of sufficient quality for clarity?

Reviewer #1: The analysis matches the methods section and are clearly presented. The figures are of great quality. Please include in this section what VISTA means instead of in the discussion section. Please include if any of the patients included had comorbidities (HIV, malnutrition, diabetes, etc.).

Reviewer #2: The analytical approach is appropriate and robust for the study design; however, the exploratory nature and limited sample size should be more explicitly acknowledged when interpreting the findings.

Reviewer #3: Could be improved. See detailed comments.

**********

**Conclusions**

-Are the conclusions supported by the data presented?

-Are the limitations of analysis clearly described?

-Do the authors discuss how these data can be helpful to advance our understanding of the topic under study?

-Is public health relevance addressed?

Reviewer #1: Please replace “chemotherapeutic agents” with antibiotics in line 314. Please replace “benign” with a more appropriate term.

Reviewer #2: The conclusions are generally supported by the data, particularly the identification of a peri-grain immune microenvironment enriched in CD66b⁺ARG1⁺VISTA⁺ cells. The limitations are acknowledged but could be more explicitly discussed, especially regarding sample size and potential bias.

Reviewer #3: Somehow. See detailed comments.

**********

**Editorial and Data Presentation Modifications?**

Use this section for editorial suggestions as well as relatively minor modifications of existing data that would enhance clarity. If the only modifications needed are minor and/or editorial, you may wish to recommend “Minor Revision” or “Accept”.

Reviewer #1: Minor revision

Reviewer #2: No further suggestions

Reviewer #3: As a neglected tropical disease, effective treatment of mycetoma requires a better understanding regarding the pathophysiology and virulence factors driving this disease. Therefore, the concept behind this manuscript and employing multiplex microscopy as well as spatial proteome analysis could address an interesting question on 1) the architecture and positioning of immune cells near the granuloma structure and 2) effector proteins shaping the immune cell response. Despite the importance and novelty, a few considerations and some experimental set-ups are required to tackle the speculations.

1. CD66b is a degranulation marker expressed by both neutrophils and eosinophils. Since eosinophils are long known to be involved and drive anti-inflammatory responses, it would be prudent that authors differentiate the two using microscopy. If neutrophils, therefore the near ROI should be CD15+/CD16+/CD14- and if eosinophils sigelc-8, CCR3, and IL-5Rα should be positive.

2. Authors are required to provide further proof that indeed the environment is immunosuppressive given that the major outcome of this manuscript, which is indeed interesting, is that the near ROI is an immunosuppressive niche. Therefore, I would suggest determining the expression of IL4, IL13, and IL14 either using ELISA or using microscopy. The latter might be more interesting since it could show if those cytokines are colocalized with CD66b.

3. Although it’s possible to infer that this immunosuppressive response is driven by the pathogen, authors should note that 1) the involvement of mycetoma in driving this response has yet to be fully confirmed and validated, and 2) this might be an appropriate immune response to the tissue destruction created by the grain structure and therefore maintaining homeostasis and healing. The latter is also possible since based on Figure 2, the expression of fibronectin and SMA are significantly lower in near compared to far ROI. Therefore, immunosuppressive immune response could be mounted to restore the shortage and restore homeostasis. But it also may imply that the proper immune cells restoring the homeostasis is not sufficient near ROI, which could question the very nature of the finding. That is why also inclusion of the alternatively activated cytokines- IL4/IL13/IL14- is important in this context.

4. Why authors did not include inflammatory proteins, including IL6, IL1β, IFNγ, and TNFα?

5. How the anti-inflammatory responses of actinomycetoma are reconciled with previous data suggesting that this complication is inflammatory as started by authors in the beginning of discussion?

Minor points

1. It would be prudent to provide microscopic images of all patients in supplementary files.

2. In the beginning of discussion section, since the data is premature, it is not recommended to mention that this CD66b hallmark of mycetoma is pathogenic, unless well-designed immunological studies are conducted to unravel this notion. The same is true for lines 364-366.

**********

**Summary and General Comments**

Use this section to provide overall comments, discuss strengths/weaknesses of the study, novelty, significance, general execution and scholarship. You may also include additional comments for the author, including concerns about dual publication, research ethics, or publication ethics. If requesting major revision, please articulate the new experiments that are needed.

Reviewer #1: Please avoid including the results or conclusions in the introduction section.

Please clearly state the objectives on the last paragraph of the introduction.

In the methods include country and city of the devices, software, and stains employed.

The design is appropriate and the population clearly stated. The sample size is small but that would be expected for such a rare disease. Ethical standards were followed. The statistical analysis is complex but adequate.

The analysis matches the methods section and are clearly presented. The figures are of great quality. Please include in this section what VISTA means instead of in the discussion section. Please include if any of the patients included had comorbidities (HIV, malnutrition, diabetes, etc.).

Please replace “chemotherapeutic agents” with antibiotics in line 314. Please replace “benign” with a more appropriate term.

Reviewer #2: The study objectives are generally clear, aiming to characterize the spatial immune microenvironment in mycetoma; however, a clearly defined, testable primary hypothesis is lacking, and the work is better framed as exploratory. The study design is appropriate for spatial proteomic analysis, but the inclusion of only 11 out of 28 patients based on grain visibility and the use of manually selected ROIs introduce potential selection bias that should be more explicitly addressed. Ethical and regulatory requirements appear to be adequately met, with appropriate approvals and informed consent obtained.

The analytical approach is appropriate and robust for the study design; however, the exploratory nature and limited sample size should be more explicitly acknowledged when interpreting the findings.

The conclusions are generally supported by the data, particularly the identification of a peri-grain immune microenvironment enriched in CD66b⁺ARG1⁺VISTA⁺ cells. The limitations are acknowledged but could be more explicitly discussed, especially regarding sample size and potential bias.

Reviewer #3: As a neglected tropical disease, effective treatment of mycetoma requires a better understanding regarding the pathophysiology and virulence factors driving this disease. Therefore, the concept behind this manuscript and employing multiplex microscopy as well as spatial proteome analysis could address an interesting question on 1) the architecture and positioning of immune cells near the granuloma structure and 2) effector proteins shaping the immune cell response. Despite the importance and novelty, a few considerations and some experimental set-ups are required to tackle the speculations.

1. CD66b is a degranulation marker expressed by both neutrophils and eosinophils. Since eosinophils are long known to be involved and drive anti-inflammatory responses, it would be prudent that authors differentiate the two using microscopy. If neutrophils, therefore the near ROI should be CD15+/CD16+/CD14- and if eosinophils sigelc-8, CCR3, and IL-5Rα should be positive.

2. Authors are required to provide further proof that indeed the environment is immunosuppressive given that the major outcome of this manuscript, which is indeed interesting, is that the near ROI is an immunosuppressive niche. Therefore, I would suggest determining the expression of IL4, IL13, and IL14 either using ELISA or using microscopy. The latter might be more interesting since it could show if those cytokines are colocalized with CD66b.

3. Although it’s possible to infer that this immunosuppressive response is driven by the pathogen, authors should note that 1) the involvement of mycetoma in driving this response has yet to be fully confirmed and validated, and 2) this might be an appropriate immune response to the tissue destruction created by the grain structure and therefore maintaining homeostasis and healing. The latter is also possible since based on Figure 2, the expression of fibronectin and SMA are significantly lower in near compared to far ROI. Therefore, immunosuppressive immune response could be mounted to restore the shortage and restore homeostasis. But it also may imply that the proper immune cells restoring the homeostasis is not sufficient near ROI, which could question the very nature of the finding. That is why also inclusion of the alternatively activated cytokines- IL4/IL13/IL14- is important in this context.

4. Why authors did not include inflammatory proteins, including IL6, IL1β, IFNγ, and TNFα?

5. How the anti-inflammatory responses of actinomycetoma are reconciled with previous data suggesting that this complication is inflammatory as started by authors in the beginning of discussion?

Minor points

1. It would be prudent to provide microscopic images of all patients in supplementary files.

2. In the beginning of discussion section, since the data is premature, it is not recommended to mention that this CD66b hallmark of mycetoma is pathogenic, unless well-designed immunological studies are conducted to unravel this notion. The same is true for lines 364-366.

**********

PLOS authors have the option to publish the peer review history of their article (what does this mean?). If published, this will include your full peer review and any attached files.

If you choose “no”, your identity will remain anonymous but your review may still be made public.

**Do you want your identity to be public for this peer review?**  For information about this choice, including consent withdrawal, please see our Privacy Policy.

Reviewer #1: No

Reviewer #2: No

Reviewer #3: **Yes:** Amir Arastehfar

**Figure resubmission:**

While revising your submission, we strongly recommend that you use PLOS’s NAAS tool (https://ngplosjournals.pagemajik.ai/artanalysis) to test your figure files. NAAS can convert your figure files to the TIFF file type and meet basic requirements (such as print size, resolution), or provide you with a report on issues that do not meet our requirements and that NAAS cannot fix.

-->

After uploading your figures to PLOS’s NAAS tool - https://ngplosjournals.pagemajik.ai/artanalysis, NAAS will process the files provided and display the results in the "Uploaded Files" section of the page as the processing is complete. If the uploaded figures meet our requirements (or NAAS is able to fix the files to meet our requirements), the figure will be marked as "fixed" above. If NAAS is unable to fix the files, a red "failed" label will appear above. When NAAS has confirmed that the figure files meet our requirements, please download the file via the download option, and include these NAAS processed figure files when submitting your revised manuscript.-->

**Reproducibility:**

To enhance the reproducibility of your results, we recommend that authors of applicable studies deposit laboratory protocols in protocols.io, where a protocol can be assigned its own identifier (DOI) such that it can be cited independently in the future. Additionally, PLOS ONE offers an option to publish peer-reviewed clinical study protocols. Read more information on sharing protocols at https://plos.org/protocols?utm_medium=editorial-email&utm_source=authorletters&utm_campaign=protocols

-->

# Author comment: Author response to Decision Letter 1

**DOI:** 10.1371/journal.pntd.0014230.r002

6 Aug 2026

Attachment Submitted filename: Osman et al_PNTD_Response to reviewers.docx

# Editor report: Decision Letter 1

**DOI:** 10.1371/journal.pntd.0014230.r003

**Authors:** Joseph Vinetz

10 Aug 2026

Dear Prof. Kaye,

We are pleased to inform you that your manuscript 'A conserved grain-associated immunosuppressive niche in Sudanese patients with mycetoma.' has been provisionally accepted for publication in PLOS Neglected Tropical Diseases.

Before your manuscript can be formally accepted you will need to complete some formatting changes, which you will receive in a follow up email. A member of our team will be in touch with a set of requests.

Please note that your manuscript will not be scheduled for publication until you have made the required changes, so a swift response is appreciated.

IMPORTANT: The editorial review process is now complete. PLOS will only permit corrections to spelling, formatting or significant scientific errors from this point onwards. Requests for major changes, or any which affect the scientific understanding of your work, will cause delays to the publication date of your manuscript.

Should you, your institution's press office or the journal office choose to press release your paper, you will automatically be opted out of early publication. We ask that you notify us now if you or your institution is planning to press release the article. All press must be co-ordinated with PLOS.

Thank you again for supporting Open Access publishing; we are looking forward to publishing your work in PLOS Neglected Tropical Diseases.

Best regards,

Joseph M. Vinetz

Section Editor

PLOS Neglected Tropical Diseases

Joseph Vinetz

Section Editor

PLOS Neglected Tropical Diseases

Shaden Kamhawi

co-Editor-in-Chief

PLOS Neglected Tropical Diseases

orcid.org/0000-0003-4304-636XX

Paul Brindley

co-Editor-in-Chief

PLOS Neglected Tropical Diseases

orcid.org/0000-0003-1765-0002

***********************************************************

p.p1 {margin: 0.0px 0.0px 0.0px 0.0px; line-height: 16.0px; font: 14.0px Arial; color: #323333; -webkit-text-stroke: #323333}span.s1 {font-kerning: none

# Editor report: Acceptance letter

**DOI:** 10.1371/journal.pntd.0014230.r004

**Authors:** Joseph Vinetz

Dear Prof. Kaye,

We are delighted to inform you that your manuscript, "A conserved grain-associated immunosuppressive niche in Sudanese patients with mycetoma.," has been formally accepted for publication in PLOS Neglected Tropical Diseases.

We have now passed your article onto the PLOS Production Department who will complete the rest of the publication process. All authors will receive a confirmation email upon publication.

The corresponding author will soon be receiving a typeset proof for review, to ensure errors have not been introduced during production. Please review the PDF proof of your manuscript carefully, as this is the last chance to correct any scientific or type-setting errors. Please note that major changes, or those which affect the scientific understanding of the work, will likely cause delays to the publication date of your manuscript. Note: Proofs for Front Matter articles (Editorial, Viewpoint, Symposium, Review, etc...) are generated on a different schedule and may not be made available as quickly.

Soon after your final files are uploaded, the early version of your manuscript will be published online unless you opted out of this process. The date of the early version will be your article's publication date. The final article will be published to the same URL, and all versions of the paper will be accessible to readers.

For Research Articles, you will receive an invoice from PLOS for your publication fee after your manuscript has reached the completed accept phase. If you receive an email requesting payment before acceptance or for any other service, this may be a phishing scheme. Learn how to identify phishing emails and protect your accounts at https://explore.plos.org/phishing.

Thank you again for supporting open-access publishing; we are looking forward to publishing your work in PLOS Neglected Tropical Diseases.

Best regards,

Shaden Kamhawi

co-Editor-in-Chief

PLOS Neglected Tropical Diseases

Paul Brindley

co-Editor-in-Chief

PLOS Neglected Tropical Diseases
